# A machine learning method to monitor China’s AIDS epidemics with data from Baidu trends

**DOI:** 10.1371/journal.pone.0199697

**Published:** 2018-07-11

**Authors:** Yongqing Nan, Yanyan Gao

**Affiliations:** School of Economics and Management, Southeast University, Nanjing, Jiangsu, China; The University of Hong Kong, CHINA

## Abstract

**Background:**

AIDS is a worrying public health issue in China and lacks timely and effective surveillance. With the diffusion and adoption of the Internet, the ‘big data’ aggregated from Internet search engines, which contain users’ information on the concern or reality of their health status, provide a new opportunity for AIDS surveillance. This paper uses search engine data to monitor and forecast AIDS in China.

**Methods:**

A machine learning method, artificial neural networks (ANNs), is used to forecast AIDS incidences and deaths. Search trend data related to AIDS from the largest Chinese search engine, Baidu.com, are collected and selected as the input variables of ANNs, and officially reported actual AIDS incidences and deaths are used as the output variable. Three criteria, the mean absolute percentage error, the root mean squared percentage error, and the index of agreement, are used to test the forecasting performance of the ANN method.

**Results:**

Based on the monthly time series data from January 2011 to June 2017, this article finds that, under the three criteria, the ANN method can lead to satisfactory forecasting of AIDS incidences and deaths, regardless of the change in the number of search queries.

**Conclusions:**

Despite the inability to self-detect HIV/AIDS through online searching, Internet-based data should be adopted as a timely, cost-effective complement to a traditional AIDS surveillance system.

## Introduction

China’s first AIDS case was reported in 1985, and the HIV epidemic in China started to spread among intravenous drug users in the late 1980s. Since 1995, China has experienced a rapid increase in the number of cases of HIV/AIDS caused by paid plasma donors in central China. In recent years, especially since 2007, sexually transmitted AIDS cases have steadily increased and consist of over half the reported HIV/AIDS infections [1,2].

According to the 2016 Statistical Bulletin of China’s National Health and Family Planning Commission, AIDS incidences contribute 1.84% of the country’s total officially announced infectious diseases, while AIDS deaths account for 78.42% of the total deaths caused by those diseases, and the ratio of AIDS deaths to AIDS incidences is as high as 25.92%. In other words, HIV/AIDS has become the leading cause of mortality from infectious diseases. Faced with the rapid spread of AIDS, in December 2003 China initiated its ‘Four Free and One Care’ policy: free ARV drugs, free prevention of mother-to-child transmission, free voluntary counselling and testing, free schooling for children orphaned by AIDS, and care for people living with HIV/AIDS. However, there are still challenges and bottleneck areas regarding the timely and accurate surveillance and effective implementation of the AIDS control programme, including the lack of tools to measure the epidemic trends accurately, the inability to monitor and predict treatment effects in a timely manner, and the inability to provide effective prevention and treatment against HIV [1].

Various indicators have been explored to estimate the actual number of patients affected by a given illness, including school and workforce absentee records [3], over-the-counter medication sales [4], phone calls and visits to doctors and hospitals [5], and news reports [6]. The monitoring based on these indicators in the extant literature has some drawbacks, such as data inaccuracy and time delay, due to too small a sample size and time-consuming information processing. The data on epidemic activities reported by China’s National Centre for Disease Control are usually lagged from two to six months. Nowadays, some novel approaches using informal sources, such as social media data [7] and search query data [8], are being developed for timely disease surveillance and forecasting. However, literature using online searching data to estimate AIDS remains absent, which is perhaps mainly due to the fact that HIV/AIDS can only be detected by professionals with laboratory instruments. According to the definition by the World Health Organization (WHO) [9], a case of HIV infection is defined as an individual with HIV infection regardless of the clinical stage (including severe or stage 4 clinical disease, also known as AIDS), confirmed by laboratory criteria. Specifically, there are three criteria for HIV diagnosis (including AIDS): (1) immunological criteria used for a child younger than 5 with confirmed HIV infection: for children < 12 months, %CD4+ <30; for those aged 12–35 months, %CD4+ <25; and for those aged 36–59 months, %CD4+ <20; (2) clinical criteria used for children and adults with confirmed HIV infection: definitive or presumptive diagnosis of any stage 3 or stage 4 condition; and (3) immunological criteria used for children and adults 5 years or older with confirmed HIV infection: a CD4 count less than 350 per mm^3^ of blood. Any one of the above three criteria can be diagnosed as advanced HIV, which includes AIDS [9].

With the rapid diffusion and adoption of the Internet, networks have gradually become an essential information resource for both physicians and patients [10]. Physicians may adopt search engines to access health care information, while patients may also use them to search for keywords that reflect their symptoms or to aid initial self-diagnosis [11]. In addition, ‘big data’ based on online search information can provide health authorities with the location, timing, and intensity of an epidemic and alert the public and disease control departments (DCDs) to a lurking health threat. Although there are some limitations, such as the Internet being inaccessible in some rural and western regions and noisy irrelevant information, web search trend data have been explored as a low-cost, nearly real-time approach to monitoring disease activity. Recently, Internet search query data have been shown to be a promising surveillance method for specific and sensitive monitoring of infectious diseases, harnessing data that are easily processed, aggregated, and visualized in near real time [12]. This paper aims to forecast and monitor AIDS deaths and incidences with a machine learning method, artificial neural networks (ANNs), in which monthly official reports on AIDS incidences and deaths are used as the output variable and online search query data are used as inputs. Indeed, the volume of Baidu searches for HIV- and AIDS-related terms may not reflect the HIV incidence or prevalence rates at the population level. There are many reasons why people may search for HIV- and AIDS-related terms other than personal health-seeking behaviours, including: student assignments; health information seeking by medical staff, researchers, friends, and relatives of PLHIV (people living with HIV); and searches by the general public in response to media reporting about HIV/AIDS. However, it is also the case that, at the population level, in the case of HIV and other communicable diseases, researchers have consistently identified positive spatial and temporal correlations between specific disease-related searches and laboratory-confirmed disease incidence and prevalence, as shown by extant studies [8,13,14]. Our findings show that search trend surveillance can be a novel, cost-effective complement to traditional disease surveillance systems and enable DCDs or the public to achieve accurate and timely AIDS monitoring.

## Material and method

### Data sources

AIDS data: We use two indicators to measure the actual AIDS outcome: monthly AIDS incidences and AIDS deaths. The data are collected from the monthly report of the National Health and Family Planning Commission of China (http://www.moh.gov.cn/), ranging from January 2011 to June 2017. The data are aggregate data, open to the public, and thus display no personal information.

Search trend data: The Internet and online search engines have been broadly used in China. A report issued by the China Internet Network Information Centre indicated that there are 566 million Chinese search engine users, and the Internet usage rate reached 82.3% at the end of 2015 [15]. We select the Baidu search trend, the weighted sum of the search frequency of each keyword, as the main data source, since Baidu.com (https://www.baidu.com/) is the largest Chinese search engine, holding the largest market share in China with a market penetration rate of 93.1%, followed by Google with a market penetration rate of only 18.0%. We obtain all the relevant search query volumes from the Baidu Index (http://index.baidu.com/), which is publicly released by Baidu.com daily. The volumes from both PC users and mobile users are integrated to make full use of the information available. Since the official AIDS data are reported monthly, we aggregate the daily search volumes into monthly ones.

### Search queries

Since thousands of keywords are potentially related to AIDS, we perform the following three procedures to choose the search queries and mine the Baidu search trend data: determine the query base, extract and rectify the data, and filter the queries.

Query base: Previous studies have generally selected the names or clinical symptoms of target diseases as their core keywords but have not mined relevant queries further [16,17]. Since there are notable differences in users’ search habits, preferences, and concerns, their practice of choosing relevant search queries may be arbitrary and omit some crucial search queries. Therefore, we first choose AIDS as the basic search query (in Chinese) and then use keyword-mining tools, Webmaster tools (http://tool.chinaz.com/), Qucha (http://www.7c.com/keyword/), and Aizhan (http://ci.aizhan.com/) to obtain the related queries. As a result, we obtain a query base converging to 201 search queries. After entering these keywords into the Baidu Index and dropping some queries with insufficient search volumes, we finally acquire a search query library (SQL) with 195 search queries regarding AIDS/HIV. These related search queries recommended by mining tools cover almost all the aspects of AIDS causes, symptoms, treatment, prevention, and so on and thus contain more comprehensive information on AIDS and AIDS-related activities.

**Fig 1:**
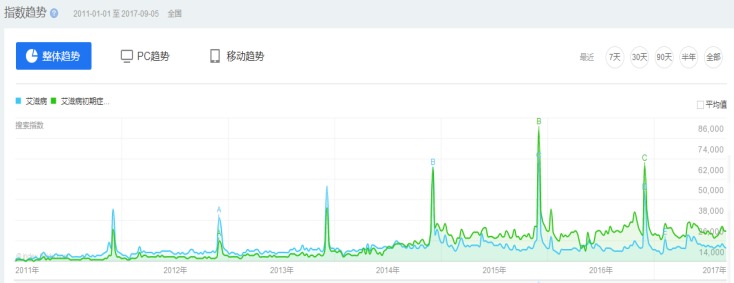
Baidu search trends with ‘AIDS (艾滋病)’ and ‘initial symptoms of AIDS (艾滋病初期症状)’ as search queries.

Data extraction and rectification: We extract candidate data series from the Baidu Index (https://index.baidu.com/) based on the above-defined search keywords. Fig 1 shows the Chinese query interface of the Baidu Index with ‘AIDS(艾滋病)’ and ‘initial symptoms of AIDS (艾滋病初期症状)’ as search queries, for which we find similar trends from 2011 to 2017. We develop a Java-based crawler to extract the daily search data from January 2011 to June 2017 through the trends, like Fig 1, since the Baidu Index does not release the data behind the search trends.

Query filtering: More search queries do not necessarily assure better-fitting results, and the marginal contribution of adding terms to a ‘saturated’ model is limited but costly [13]. Here we filter search queries with the Pearson correlation coefficient (PCC) between the AIDS incidences (or AIDS deaths) and the monthly search volumes of each search query. Meanwhile, if too large a PCC threshold is chosen, some valuable queries will be removed, which may reduce the forecasting performance of the machine learning method that we use. In our case, if the PCC threshold is set at 0.65, only two search queries are left, too few to measure searching behaviours regarding HIV/AIDS. When forecasting influenza using search queries, 0.4 has been used as the PCC threshold to filter those search queries [18].

Therefore, we first adopt 0.5 as the PCC threshold value to select search queries. If one search query volume’s PCC with AIDS incidences or deaths is larger than 0.5, this query is included in the list of predictors. As a result, 14 queries are filtered out to forecast AIDS incidences and 31 queries to forecast AIDs deaths, which are listed in S1 and S2 Tables, respectively. As an example, S1 Fig also shows that the aggregate monthly search volume of ‘Regulation on the Prevention and Treatment of AIDS’ and the official AIDS statistics display similar contemporaneous trends over time. To gauge the sensitivity of the forecasting performance, the PCC threshold value is increased by 0.05 to 0.65, leaving fewer search queries but a higher PCC of these queries with AIDS incidences and deaths.

### Method

**Fig 2:**
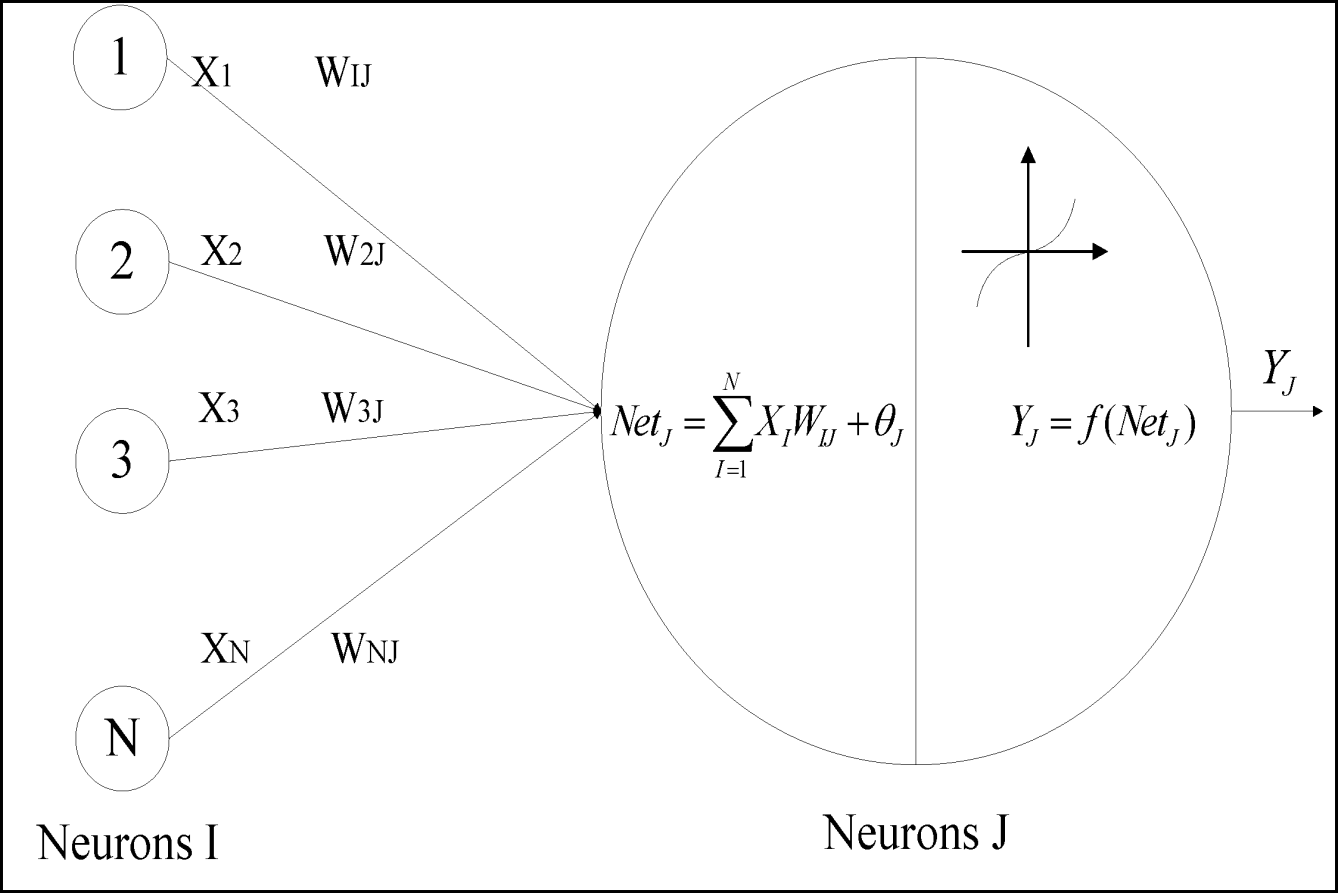
The neuron model of ANNs.

**Fig 3:**
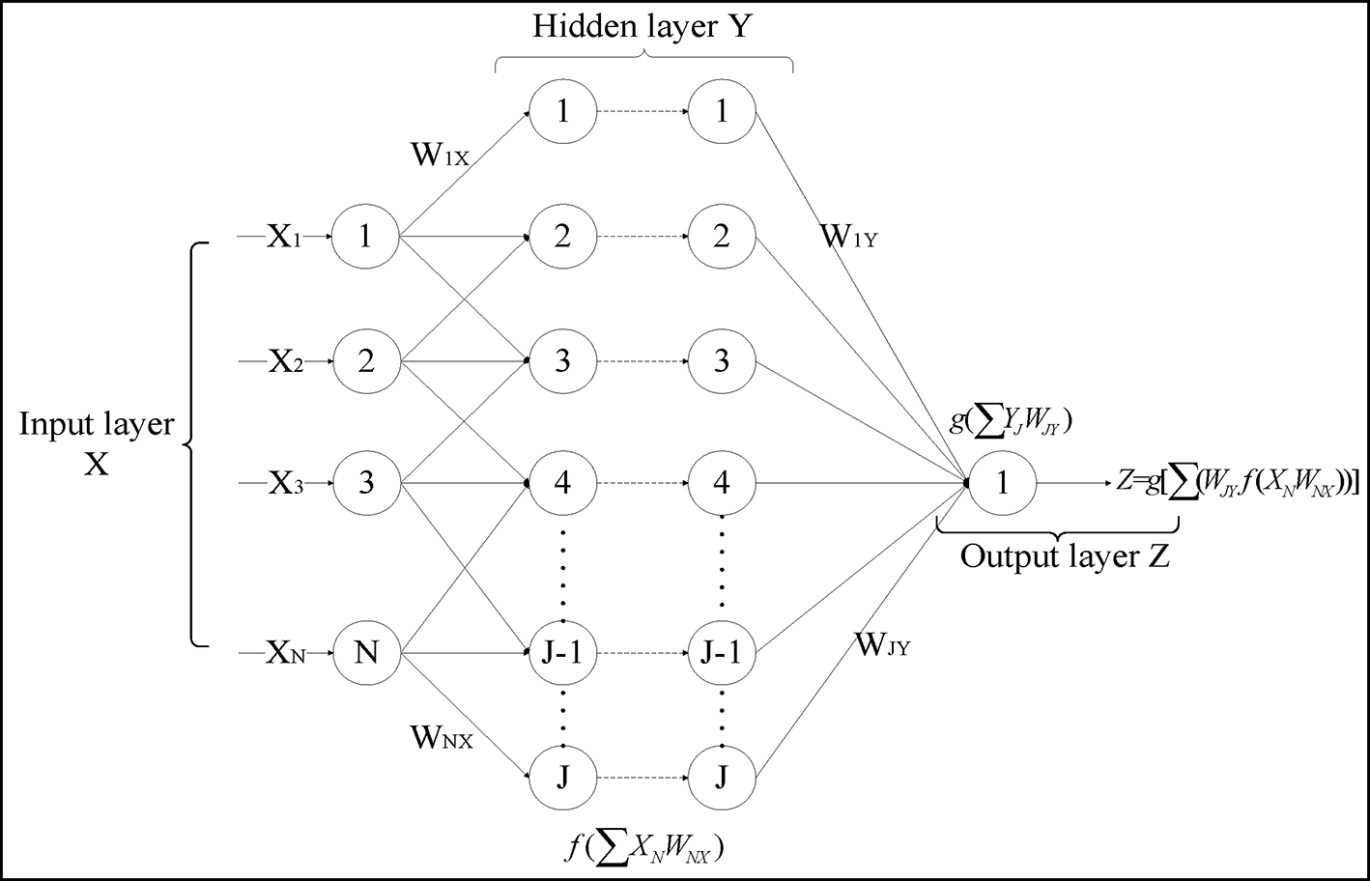
MLP feed forward ANNs’ architecture.

A machine learning method, artificial neural networks (ANNs), is used to forecast AIDS incidences and deaths in the next one to six months, for which the input variables are the search queries from the Baidu trend data and the output variables are the officially reported actual AIDS incidences and deaths. ANNs are branches of artificial intelligence developed in the 1950s with the aim of imitating the biological brain architecture, which consists of many simple neural cells [19]. ANNs are parallel-distributed systems made of many interconnected nonlinear processing elements (PEs) called neurons [20]. The model of an artificial neuron is shown in Fig 2. Neuron *j* is the sum of input signals *x*_*i*_, weighted by connection weights *w*_*ij*_ from neighbouring neurons. These weighted signals constitute the neuron’s net input *net*_*j*_. Then, the activation threshold *ϑ*_*j*_ is added to the net input. After the activation function *f* (.) is further applied to the net input, the output value *y*_*j*_ can finally be computed and sent to other neurons. A multilayer perceptron (MLP) model is one of the most widely used nonlinear, nonparametric ANNs. MLP networks consist of an input layer, one or more hidden layers, and an output layer. The architecture of an MLP model is shown in Fig 3. For more details about the ANNs and MLP, readers can refer to the book written by Tadeusiewicz [21].

The relationship between Internet query searching and actual HIV/AIDS epidemics may be influenced by many factors, such as the regional Internet penetration rate, the health care-seeking behaviour of Internet users, or the media’s influence power. In other words, the relationship may be nonlinear, and the linear regression model may not fit it well. Since no specific assumption needs to be made, an MLP model of ANNs is also used in this paper. In terms of hidden layers and nodes, a one-hidden-layer BP network type is used, and the number of hidden nodes is determined by trial and error [22]. Moreover, the Levenberg–Marquardt backpropagation method is adopted to update the weight and bias states in each iteration, and the MATLAB^@^ Neural Networks Toolbox is used to run the MLP model. To conduct the learning process of neural networks, the MLP model randomly divides the data pairs into three subsets–the training set, the validation set, and the test set–which are sets accounting for 80%, 10%, and 10% of the observations, respectively. To accelerate the process of convergence, all the inputs and outputs are normalized by *X*_new_ = 2[(*x*-*x*_min_)/(*x*_max_-*x*_min_)]-1, where *x* denotes either any input or the output.

### Forecasting performance

To evaluate the forecasting performance of ANNs with AIDS search trend data, we employ three criteria: the mean absolute percentage error (MAPE), the root mean squared percentage error (RMSPE), and the index of agreement (IA). They are given as follows:

$$MAPE=\frac{1}{n}\sum_{i=1}^{n}\left(\left|y_{i}‑{\hat{y}}_{i}\right|/y_{i}\right)$$ (1)

$$RMSPE=\sqrt{\frac{1}{n}\sum_{i=1}^{n}{\left((y_{i}‑{\hat{y}}_{i})/y_{i}\right)}^{2}}$$ (2)

$$IA=1‑\sum_{i=1}^{n}(y_{i}‑{\hat{y}}_{i}{)}^{2}/(\sum_{i=1}^{n}\left(|y_{i}‑y_{ave}\right|+{\left|{\hat{y}}_{i}‑y_{ave}|\right)}^{2})$$ (3)

where *n* is the total period, *y*_*i*_ is the actual value, and ${\hat{y}}_{i}$ denotes the predicted value from the MLP model of ANNs. The MAPE and RMSPE measure the difference between the real AIDS indicators and their predicted value estimated from the MLP model, and in an ideal case they take the value of zero. Thus, the smaller the value is, the better the forecasting performance is. Meanwhile, the IA is a dimensionless index within the range of 0–1, and IA = 1 indicates perfect agreement between predictions and observations, that is, perfect forecasting. In practice, the extant studies have broadly used 0.05 as the threshold value of the MAPE and RMSPE and 0.6 as the threshold value of the IA for a qualified forecasting performance [23–26].

## Results

The forecasting performances of the MLP model of ANNs for AIDS are calculated on the basis of the test set, which accounts for 10% of all the observations, and the full set, that is, all the observations. In this section, the 80% training set is used to train the networks, and the 10% test set and the full set are used to evaluate the forecasting performance of the networks trained with the training set. Table 1 reports the forecasting performance of the MLP model of ANNs in the test set and the number of neurons in the input and output layers under different PCC threshold values. Each scenario is examined with 1,000 cycles of training and learning in the training set. The results show that, when the PCC threshold value is set at 0.6, the MLP model with the topology structure is (4:4:1), that is, four nodes in the input layer, four neurons in the hidden layer, and one node in the output layer. This produces the best forecasting of AIDS incidences in the test set and leads to the best forecasting of AIDS deaths in the test set with the topology structure of (18:18:1). It should also be noted that the forecasting performance is robust to the change in PCC threshold values and is very good, since both the MAPE and the RMSPE are smaller than 0.05 and the IA is larger than 0.68, all representing a highly accurate prediction [23–25].

**Table 1:** Forecasting performance of the MLP model of ANNs for AIDS in the test set.

Parameters	AIDS incidences				AIDS deaths			
PCC threshold value	0.50	0.55	0.60	0.65	0.50	0.55	0.60	0.65
Number of neurons in input layer	14	8	4	2	31	21	18	13
Number of neurons in hidden layer	25	8	4	2	32	21	18	13
MAPE	0.0157	0.0159	0.0248	**0.0149**	0.0342	0.0242	**0.0180**	0.0181
RMSPE	0.0162	0.0054	**7.0625E-05**	0.0017	0.0027	0.0040	**2.2963E-06**	0.0243
IA	0.8103	0.8363	**0.8707**	0.8620	0.6843	**0.8040**	0.7290	0.7914

Note: The optimal results are highlighted in bold.

After training the ANNs with the training set to realize the best forecasting of AIDS in the test set, we further use the optimal networks to predict AIDS in the full set to determine whether the forecasting performance remains unchanged. Table 2 reports the full-set forecasting performance. The results are reassuring. When the PCC threshold value is set at 0.6 and 0.5, the well-trained MLP model leads to the best forecasting of AIDS incidences and deaths, respectively, according to the MAPE and the RMSPE for AIDS incidence forecasting and the MAPE and the IA for AIDS death forecasting.

**Table 2:** Forecasting performance of the MLP model of AIDS in the full set.

Parameters	AIDS incidences				AIDS deaths			
PCC threshold value	0.50	0.55	0.60	0.65	0.50	0.55	0.60	0.65
Number of neurons in input layer	14	8	4	2	31	21	18	13
Number of neurons in hidden layer	25	8	4	2	32	21	18	13
MAPE	0.0014	0.0014	**0.0012**	0.0016	**8.8229e-04**	0.0011	0.0034	0.0013
RMSPE	**8.7619e-04**	0.0039	0.0012	0.0022	00018	**0.0012**	0.0202	0.0031
IA	0.8596	0.8779	**0.8985**	0.8465	**0.9232**	0.9192	0.5868	0.8055

Note: The optimal results are highlighted in bold.

**Fig 4:**
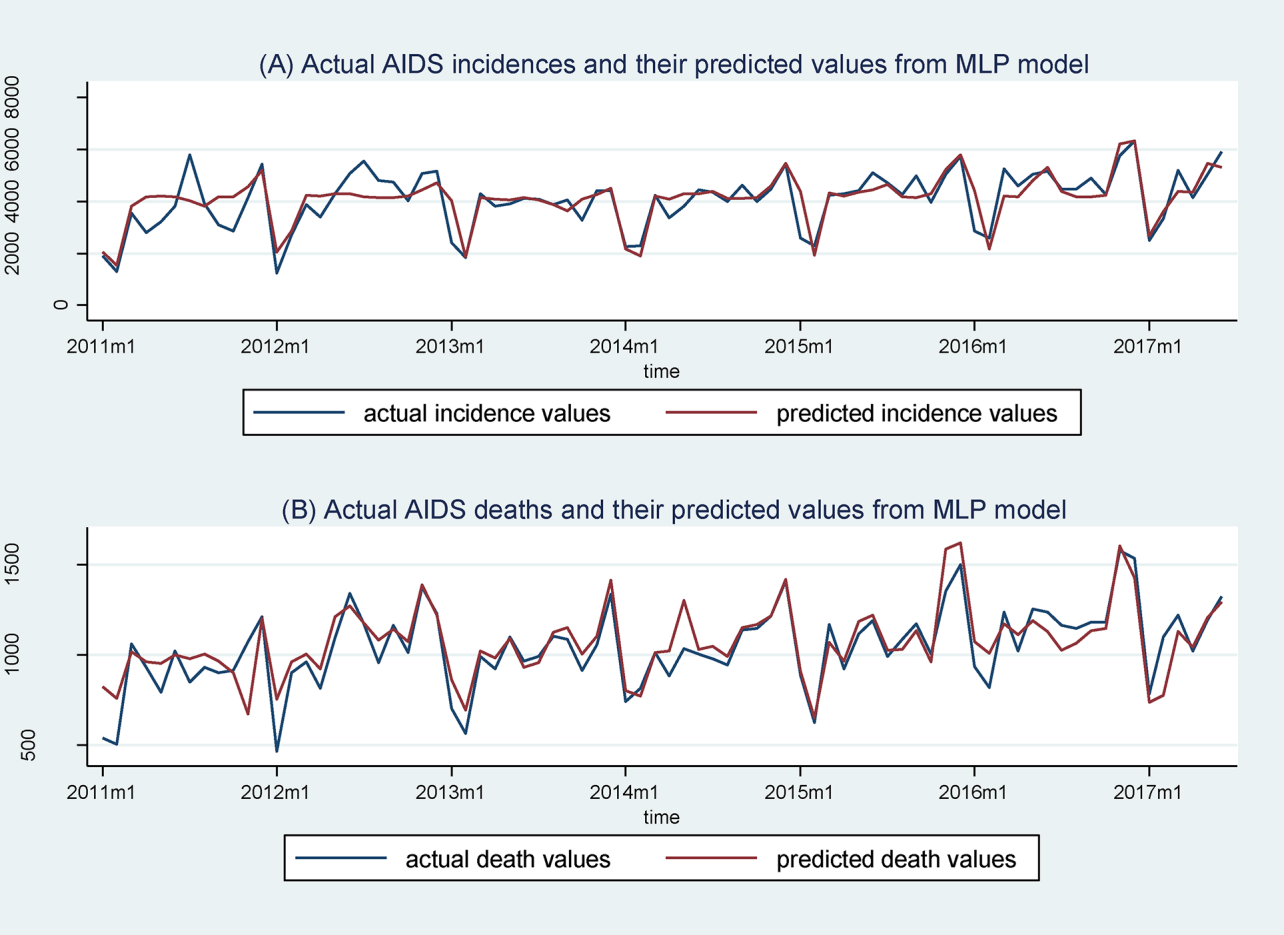
Forecasting AIDS with a well-trained MLP model. Note: The predicted values are from a well-trained MLP model with PCC threshold values of 0.6 and 0.5, which provide the best forecasting to actual AIDS incidences and deaths in the full sample, respectively.

Fig 4 displays the trends of actual AIDS incidences and deaths and their predicted values from the MLP model with the best forecasting performance over time. The actual AIDS incidences and deaths are in blue, while their predicted values from the MLP model are in red. It is easy to tell that the two variables nearly overlap over time, exhibiting almost the same trends and being consistent with Table 1 as accurate forecasting. This best-fitting performance can also be shown by graphing two-way scatters of the AIDS statistics and their predicted values from the MLP model, in which the predicted values can attain the best forecasting to the actual values of AIDS incidences when the PCC threshold value is 0.6 and to those of AIDS death when that threshold value is 0.5 (see S2 Fig).

Meanwhile, we also report the optimal prediction horizons of AIDS incidences and deaths resulting from the MLP model of the ANN method. First, the optimal net is obtained based on the evaluation of the test set, then the values of the MAPE/RMSPE/IA and the topology structures are determined from recursive forecasts for the next one to six months. The forecasting performances under different horizons are reported in S3 Table. We find that, under the MAPE/IA criteria, the MLP model can best forecast AIDS incidences in the next second month, but, under the RMSPE criterion, it can best forecast AIDS incidences in the next fifth month. In terms of AIDS deaths, both the MAPE and the RMSPE also indicate that the model produces the best forecasting in the next month, and the IA indicates the best forecasting in the next third month. However, despite the changes in the three criteria, the MLP model performs quite well in forecasting both AIDS incidences and AIDS deaths in the next first to fourth months, since all three evaluation criteria have acceptable values: smaller than 0.05 for the MAPE and RMSPE and larger than 0.7 for the IA. Better forecasting performance of shorter horizons can also be observed in S3 Fig.

## Discussion and conclusion

### Discussion

The Internet provides almost limitless real-time, cheap data on consumers’ searching behaviours, while ANNs provide solid novel methods for accurate forecasting. However, there might be several limitations to our study. First, the Baidu search trend data regarding AIDS might lack representation, since they are restricted to users of the Baidu search engine. Internet access is uneven in China and between rural and urban areas. Search query data represent only part of AIDS victims’ searching behaviours and thus are just a complementary but important method for monitoring real-time AIDS incidences and deaths. On the other hand, due to social discrimination against AIDS victims, HIV-infected people are usually unwilling to report their disease or they resort to informal treatments. Besides, social ignorance regarding HIV and AIDS makes HIV carriers fail to realize their disease. As a result, the incidences and deaths may be under-reported in the official statistics.

Second, we did not compare the forecasting performance between the ANN method and the traditional time series models, such as the autoregressive and moving average, vector autoregressive, and vector error correction models, since the data contain only 78 observations and the variables are not necessarily stationary, which may reduce the credibility of the estimations produced by these time series models. However, a recent study has suggested that the ANN method can be a promising alternative to traditional linear methods [27]. The ordinary least square method also shows that the predicted values from the optimal MLP model can significantly explain actual AIDS incidences and deaths with a goodness of fit of 0.681 and 0.754, respectively (see S2(i)-(C) and S2(ii)-(A) Fig).

Third, some search behaviours may be generated by news reports, special events, or a ‘celebrity effect’ rather than actual disease activity. For example, the search volumes of some search queries, such as ‘AIDS workers are advised to rest’ and ‘NBA famous player Johnson is infected with AIDS’, are due to news reports that an AIDS victim had been fired by his employer and a famous basketballer named Johnson had been infected by HIV, respectively. However, these queries generally have a low correlation coefficient with actual AIDS incidences and deaths and were removed from the list of inputs of the MLP model. Another concern is that the sharp increase in the search trend of AIDS queries on particular days, say, 1 December 1, that is, World AIDS Day, also does not reflect the actual AIDS incidences or deaths but rather indicates people’s attention to AIDS. However, it is apparent from Fig 4 that actual AIDS incidences and deaths also increase on this day. The synchronous increases may occur because AIDS awareness activities generate more related searching behaviours; less discrimination against and greater caring for AIDS victims happen around the day as a result of the information campaign; and more knowledge about AIDS makes AIDS victims more willing to disclose their disease.

Finally, because of the lack of provincial or city-level data, we only conducted a study at the national level. China is a large country with diversified geographies, cultures, ethnicities, and population distribution. Thus, our results may have limited policy implications for how local governments can use search trends data to monitor local AIDS incidences and deaths. Moreover, we only use monthly data rather than weekly data or daily data, which are also unavailable. We admit that a study with weekly or daily data will lead to a more accurate forecasting performance on AIDS because of their merits of real-time and high-frequency observations. As an exploratory study, our framework using Baidu search trend data to forecast AIDS incidences and deaths can easily be extended to the provincial or city level.

However, despite the limitations of online search data, the ANN method, as an information-processing system inspired by biological neural networks [28], does not require any pre-assumptions regarding the relationship between the input variables and an outcome variable. It can process various fuzzy, nonlinear, noisy data through neuron simulation, memory, and association, and it processes calculation analysis using the method of self-adapting pattern recognition [29]. Moreover, three fundamental features of ANNs, that is, parallel processing, distributed memory, and adaptability, enable them to tolerate errors and noises and thereby provide robust forecasting. Thus, the ANN method can make full use of the information behind the search trends to realize satisfactory forecasting of AIDS incidences and deaths. Of course, to extend our study, it is necessary to collect data in different regions or with a longer time horizon and then compare them with traditional time series models.

### Conclusion

ANNs perform well in forecasting China’s monthly AIDS incidences and deaths with Baidu trend data. Three criteria of forecasting performance, the MAPE, the RMSPE, and the IA, all indicate that the MLP model of ANNs can result in accurate forecasting of concurrent AIDS incidences and deaths with Baidu search trend data. Moreover, the forecasting performance is relatively robust to a change in the number of search queries and forecasting horizons.

Our results have strong policy implications for both AIDS surveillance and the forecasting of other diseases. As people increasingly engage in their work and lives online, their traces on the Internet can be used to monitor their behaviours. Although HIV/AIDS can only be detected by professional laboratory instruments, potentially infected victims’ searches through search engines with keywords relating to AIDS reveal the future risk of an AIDS/HIV outbreak. Thus, AIDS/HIV surveillance based on online search queries is becoming increasingly urgent. It costs less and is more immediate than traditional surveillance based on hospital data, which are lagged by days, weeks, or even longer [30]. In our case, forecasting AIDS one to four months ahead of time could enable DCDs or the public to prioritize the detection and prevention of the spread of AIDS/HIV. As people increasingly access the Internet, search queries will become more representative and can be used to monitor other emerging and re-emerging diseases in other countries and regions with high AIDS prevalence, given that data on actual AIDS are available.

## Supporting information

S1 Fig Actual AIDS incidences and deaths, and search volumes of ‘Regulation on the Prevention and Treatment of AIDS’. (DOCX)

S2 Fig AIDS forecasting performance with the MLP model of ANNs. (DOCX)

S3 Fig Actual AIDS incidences and deaths, and predicted volumes of different horizons. (DOCX)

S1 Table Search queries and their correlation with AIDS incidences. (DOCX)

S2 Table Search queries and their correlation with AIDS deaths. (DOCX)

S3 Table Forecasting performances of different horizons. (DOCX)
