# Supplementary material for: A machine learning method to monitor China’s AIDS epidemics with data from Baidu trends
Source: PLoS One. 2018 Jul 11;13(7):e0199697. doi: 10.1371/journal.pone.0199697 (PMC6040727; doi:10.1371/journal.pone.0199697)
Supplement: S2 Fig — (DOCX) [file pone.0199697.s002.docx]

**S2 Fig.**

(i) AIDS incidences and their predicted values from MLP model

(ii) AIDS deaths and their predicted value from MLP model

Notes: All variables are stationary according to augmented dick-fuller unit roots test; y denotes actual AIDS incidences or deaths, and x denotes their predicted values; p-value are in parentheses and estimated coefficients are in the equation, which are estimated from OLS; the number of observations is 78.

Data sources: See the paper.
