# Supplementary material for: A machine learning method to monitor China’s AIDS epidemics with data from Baidu trends
Source: PLoS One. 2018 Jul 11;13(7):e0199697. doi: 10.1371/journal.pone.0199697 (PMC6040727; doi:10.1371/journal.pone.0199697)
Supplement: S3 Fig — (DOCX) [file pone.0199697.s003.docx]

**S3 Fig.**

(i) AIDS incidences and their predicted volumes of different horizons

(ii) AIDS deaths and their predicted volumes of different horizons

Data sources: See the paper.
