# Supplementary material for: A machine learning method to monitor China’s AIDS epidemics with data from Baidu trends
Source: PLoS One. 2018 Jul 11;13(7):e0199697. doi: 10.1371/journal.pone.0199697 (PMC6040727; doi:10.1371/journal.pone.0199697)
Supplement: S1 Table — (DOCX) [file pone.0199697.s004.docx]

**S1 Table****.**

| Queries in Chinese  (in English) | Pearson correlation coefficient | Queries in Chinese  (in English) | Pearson correlation coefficient |
| --- | --- | --- | --- |
| 艾滋病防治条例（Regulations on the Prevention and Treatment of AIDS） | 0.7331 | 艾滋病手抄报（Handwritten AIDS newspaper） | 0.5516 |
| 艾滋病防治知识（AIDS prevention knowledge） | 0.6566 | 如何预防艾滋病（How to prevent AIDS） | 0.5378 |
| 艾滋病的传播途径（Route of transmission of AIDS） | 0.6285 | 预防艾滋病手抄报（Handwritten anti-AIDS newspaper） | 0.5322 |
| 艾滋病宣传（AIDS campaign） | 0.6037 | 艾滋病（AIDS） | 0.5312 |
| 艾滋病病毒（AIDS virus） | 0.5952 | 什么是艾滋病（What is AIDS） | 0.5199 |
| 艾滋病起源（The origins of the AIDS） | 0.5674 | 艾滋病的起源（The origins of AIDS） | 0.5196 |
| 艾滋病的由来（The origins of the AIDS） | 0.5617 | 预防艾滋病（AIDS prevention） | 0.5026 |

Note: Baidu users search information in Chinese and corresponding translation of each Chinese Characters are listed. Some queries in Chinese are different from each other but translated into the same words in English; this table reports all search queries having a Pearson correlation coefficient with AIDS incidences larger than 0.5.
