# Supplementary material for: A machine learning method to monitor China’s AIDS epidemics with data from Baidu trends
Source: PLoS One. 2018 Jul 11;13(7):e0199697. doi: 10.1371/journal.pone.0199697 (PMC6040727; doi:10.1371/journal.pone.0199697)
Supplement: S2 Table — (DOCX) [file pone.0199697.s005.docx]

**S2 Table.**

| Queries in Chinese  (in English) | Pearson correlation coefficient | Queries in Chinese  (in English) | Pearson correlation coefficient |
| --- | --- | --- | --- |
| 艾滋病防治条例(Regulation on the Prevention and Treatment of AIDS) | 0.7987 | 怎样预防艾滋病(How to prevent AIDS) | 0.6034 |
| 艾滋病防治知识(AIDS prevention knowledge) | 0.7973 | 艾滋病宣传日(AIDS awareness day) | 0.6011 |
| 艾滋病宣传(AIDS awareness) | 0.7348 | 世界艾滋病日(World AIDS Day) | 0.599 |
| 艾滋病手抄报(Handwritten AIDS newspaper) | 0.7178 | 怎么预防艾滋病(How to prevent AIDS) | 0.5845 |
| 预防艾滋病手抄报(Handwritten anti-AIDS newspaper) | 0.6999 | 艾滋病宣传标语(AIDS awareness slogan) | 0.5774 |
| 艾滋病病毒(AIDS virus) | 0.6977 | 艾滋(AIDS/HIV) | 0.5496 |
| 如何预防艾滋病(How to prevent AIDS) | 0.6967 | 艾滋病初期症状(Initial symptoms of AIDS) | 0.546 |
| 艾滋病的传播途径(Route of transmission of AIDS) | 0.6928 | 艾滋病皮疹图片(Images of AIDS skin rashes) | 0.5239 |
| 预防艾滋病(AIDS prevention) | 0.6865 | 得了艾滋病能活多久(How long will one survive once he/she contracts HIV) | 0.52 |
| 什么是艾滋病(What is AIDS) | 0.6703 | 艾滋病患者能活多久(How long can AIDS patients survive) | 0.5125 |
| 世界艾滋病日是哪一天(Which day is World AIDS Day) | 0.6684 | 艾滋病的预防(HIV/AIDS prevention) | 0.51 |
| 艾滋病的由来(the origins of the AIDS) | 0.6654 | 河南艾滋病村(AIDS village in Henan province) | 0.51 |
| 艾滋病起源(The origins of the AIDS) | 0.6645 | 艾滋病怎么得的(How does one contract HIV) | 0.5087 |
| 艾滋病(AIDS) | 0.6455 | 中国艾滋病人数(Number of AIDS patients in China) | 0.5065 |
| 艾滋病日(AIDS Day) | 0.6355 | 艾滋病潜伏期症状(Symptoms of AIDS in incubation period) | 0.5033 |
| 艾滋病的起源(The origins of AIDS) | 0.6138 |  |  |

Notes: Baidu users search information in Chinese and corresponding translation of each Chinese Characters are listed. Some queries in Chinese are different from each other but translated into the same words in English; this table reports all search queries having a Pearson correlation coefficients with AIDS incidences larger than 0.5.
