# Supplementary material for: A machine learning method to monitor China’s AIDS epidemics with data from Baidu trends
Source: PLoS One. 2018 Jul 11;13(7):e0199697. doi: 10.1371/journal.pone.0199697 (PMC6040727; doi:10.1371/journal.pone.0199697)
Supplement: S3 Table — (DOCX) [file pone.0199697.s006.docx]

**S3 Table.**

| Forecasting horizons | | 1 month | 2 months | 3 months | 4 months | 5 months | 6 months |
| --- | --- | --- | --- | --- | --- | --- | --- |
| AIDS incidences | Structures | 4:4:1 | 4:4:1 | 4:4:1 | 4:4:1 | 4:4:1 | 4:4:1 |
|  | MAPE | 0.0020 | **0.0015** | 0.0019 | 0.0018 | 0.0019 | 0.0022 |
|  | RMSPE | 0.0060 | 0.0037 | 0.0069 | 0.0037 | **0.0018** | 0.0062 |
|  | IA | 0.6867 | **0.8793** | 0.7664 | 0.7139 | 0.7279 | 0.6736 |
| AIDS deaths | Structures | 18:21:1 | 18:19:1 | 18:18:1 | 18:18:1 | 18:18:1 | 18:18:1 |
|  | MAPE | **5.5657e-04** | 9.1476e-04 | 8.8958e-04 | 0.0014 | 0.0025 | 0.0019 |
|  | RMSPE | **3.0084e-04** | 8.4703e-04 | 9.6963e-04 | 0.0045 | 0.0028 | 4.3997e-04 |
|  | IA | 0.9043 | 0.7344 | **0.9364** | 0.7992 | 0.4900 | 0.6196 |

Notes: According to table 1, the PCC threshold value is set at 0.6 in AIDS incidences and deaths.
